# The Effect of Self-Disclosure on Loneliness in Adolescents During COVID-19: The Mediating Role of Peer Relationships

**DOI:** 10.3389/fpsyt.2021.710515

**Published:** 2021-08-19

**Authors:** Li Chen, Rui Cheng, Bo Hu

**Affiliations:** ^1^Department of Psychology, Key Laboratory of Behavior and Mental Health of Gansu Province, Northwest Normal University, Lanzhou, China; ^2^Department of Psychology, Northwest Normal University, Lanzhou, China

**Keywords:** COVID-19, adolescent, self-disclosure, loneliness, peer relationship, mediation

## Abstract

Long-term home isolation has had a certain impact on adolescents' enthusiasm for interpersonal communication and desire for self-disclosure during COVID-19. The purpose of this study was to explore the relationship between adolescents' self-disclosure and loneliness during COVID-19, and to analyze the mediating role of peer relationship in it. We conducted a cross-sectional study involving 830 Chinese adolescents (males: 47.5%, Mage14.25 years; females: 52.05%, Mage 14.19 years; Age range 12-15). Participants completed a self-reported survey that included sociodemographic, Jourard Self-Disclosure Scale, UCLA, and Peer Relationship Scale. The results showed that in the period of COVID-19, adolescents' self-disclosure affects loneliness through peer relationship, that is, the level of self-disclosure can significantly predict loneliness through peer relationship, and peer relationship plays a complete mediating role.

## Introduction

The outbreak of COVID-19 in 2019 has had a huge impact on people's lives, affecting their work, study, and travel. The pandemic has been a global health emergency, and it may have a serious impact on public health, including mental health. According to the features of its epidemic, COVID-19 is highly infectious and transmits from human-to-human with a certain incubation period (1). After finding out the feature, the Chinese government imposed urgent countermeasures to control the rapid spread of COVID-19. On January 25, 2020, the Standing Committee of the Political Bureau of the CPC Central Committee held a meeting to study the prevention and control of pneumonia in the infection, and it was suggested that such countermeasures as “preventing spread in the domestic and safeguarding imports from foreign countries, having collective quarantine for all patients, undertaking home medicine management for all close-contacting people, and working to prevent the spread of the epidemic” be adopted. The Ministry of Education proposed students should “stop attending class but keep learning” to prevent the spread in campuses. Postponing schooling and imposing home quarantine were important measures (2). Long-term family living and study would cause many negative psychological impacts for many people (such as loneliness, anxiety, depression, depression, fear, stress) because they have no face-to-face class teaching to ensure safe social distance (3). People lock themselves down comprehensively. By using facial protection and maintaining safe social distancing, people may grow more wary of strangers, and find it harder to express their feelings easily.

The COVID-19 pandemic has also changed how we interact. In many countries, people were recommended or required to socially distance. This means people were asked to either physically distance when meeting in-person (i.e., stay at least six feet apart) or stay at home (i.e., leave their home only for essential activities) (4). This change has been abrupt, with some governments initially declaring the measures unnecessary and then suddenly demanding people stay at home (5). The change has also been of uncertain lengths; the initial calls for social distancing were for a few weeks, but the social isolation has extended into several months, and the rules for social distancing seem to be ever evolving (6). And the subsequent home quarantine and social distance requirements increase the loneliness, anxiety, and negative emotion of the whole social experience (7). Teenagers are an important group in the epidemic. The Chinese Ministry of Education proposed a working layout of “stop attending class and keep learning” to ensure students' physical health, but the deep impact of the continued spread of the epidemic on their psychological status is also an issue. The suddenness, aggregation, and diffusion of the pandemic meant teenagers were confined to their homes within a short time, so they were extremely apt to have the psychology of blind obedience and credulity of false information. Long-term home quarantine has imposed a certain impact on their psychology, and some of them would face negative situations such as language barriers and negative emotions from lack of face-to-face communication and exchange, so it would seriously affect teenagers' collective resistance to physical and mental illness (8). Some researchers showed that long-term staying at home would have a bigger influence on emotion. The teenagers who are very active would be in need of direct contact with the outside world, and they would have much more psychological pressure, even triggering such serious psychological issues as anxiety and depression, due to the sense of loneliness. It was found in a domestic study that the occurrence of emotional and behavioral problems in children and adolescents is higher than that before the epidemic. Individuals under 18 years old are a high-risk population (9).

Self-disclosure is an important communication channel between the individual and the outside world, and it is a necessity for individual growth. Self-disclosure would impose an impact on an individual's social adaptation; the individual should have positive self-disclosure to have effective communication with the outside world (9). The epidemic stops the channel for people to communicate with the outside world, and individuals find it hard to have a positive self-disclosure. Against the background of the epidemic, good self-disclosure is helpful to build up good companionship and family relationships to go through the epidemic with a more positive and healthier attitude. Early adolescence is a period of social change. Companionship changes from sharing activities in childhood to the feature of spending much more time in conversation with each other (10). Adolescents would have much more communication with peers and dependence on peers. During the adolescent period, there is internal value to share their own information with friends. It was found from the result of one study that there are neurophysiological and behavioral differences when disclosing information about one's self in different depths to peers. Take girls in adolescence as an example. A task involving self-disclosure currency choice was completed while receiving functional magnetic resonance imaging. In view of behavior, teenagers give up much more money and choose to share superficial self-reference information instead of intimate information with close friends (in real life). According to neuro analysis, the areas of social cognition and emotion regulation are supported to participate in the self-disclosure of intimate information (11). Adults would give up money for the chance of disclosure, and there would be a reward center in their brains when doing so (12). Studies show that self-disclosure is beneficial for the forming and development of companionship and intimacy (13); the production of a sense of loneliness is also related to self-disclosure. In the study by (14), three-quarters of the participants thought that the production of a sense of loneliness is no ability to have self-disclosure.

For the study on self-disclosure and sense of loneliness, the scholar Imai and Imai observed the moderating effect of cross-ethnic self-disclosure on perceived ethnic bias, depression, and loneliness of foreign students; the result shows that the self-disclosure could buffer the negative effects of prejudice on loneliness and depression (15). Franzoi et al. tested the impact of teenagers' self-disclosure on the sense of loneliness with the structural equation, and the result showed that an individual's self-consciousness has an indirect relationship with the sense of loneliness through the self-disclosure with peers, which means that young people with high self-consciousness would prefer to disclose themselves with peers to reduce the sense of loneliness (16). Salono et al. (17) showed that self-disclosing to peers instead of parents is related to less loneliness. Pingxian et al. (18) also conclude from the study of the relationship between self-disclosure and sense of loneliness that there is a significant negative correlation between individual self-disclosure and sense of loneliness. The individual with high self-disclosure would have less sense of loneliness (18). Jiang et al. also make a similar conclusion in the study of the relationship between self-disclosure and loneliness (18, 19).

During the COVID-19 pandemic, Chinese student's average quarantine time was 5 months. In the area of our study, students were required to wear masks and keep a safe distance at the beginning of school and when moving and eating. Due to our government and people's concerted effort, Chinese students have had shorter quarantine periods compared to students from other countries (for instance, Brazilian adolescents have had their face-to-face classes interrupted for more than 1 year). In conclusion, COVID-19 may exacerbate the sense of loneliness, anxiety, and panic of teenagers, and it stops their chance to communicate with peers so that there is an obstacle to their self-disclosure. More importantly, the measures we have adopted during COVID-19 may impact on adolescents, such as the possible impacts on the development of social and communication skills; among other aspects, it is not conducive to the development of the adolescent brain and personality. The study discusses the impact of self-disclosure on an individual's sense of loneliness against the background of COVID-19, and the mediation of companionship. Here are the three hypotheses. (1) There is a significant correlation between teenagers' self-disclosure and loneliness during the epidemic period; (2) There is a significant negative correlation between the teenagers' peer acceptance and loneliness during the epidemic period, while there is a significant positive correlation between the peer's fear and inferiority and loneliness; (3) In the period of COVID-19, adolescents' self-disclosure affects loneliness through peer relationship, that is, the level of self-disclosure can significantly predict loneliness through peer relationship, and peer relationship plays a complete mediating role.

## Materials and Methods

### Participant and Procedure

The study was approved by the Institutional Review Board of the Northwest Normal University. Participant's parents signed their informed consent. This cross-sectional study was conducted on Chinese adolescents in the context of the COVID-19 pandemic. Data collection was conducted from June to December using an online self-reported survey (Questionnaire Star). We randomly selected 830 junior high school students in Gansu and Shandong. And they volunteered to participate in the study and completed the questionnaire. All questions related to the survey were managed using the Questionnaire Star platform and a shareable link was generated to help teens spread the survey across different online platforms. The online survey details informed consent, purpose, and inclusion and exclusion criteria for the study on the first page.

### Measurement

A self-reported and structured online survey included sociodemographic information about the participants, as well as psychological questionnaires measuring three study variables for data collection.

### Self-Disclosure Questionnaire

Self-disclosure was measured with the brief version of The Jourard Self-Disclosure Questionnaire (JSDQ); this questionnaire prepared by Jourard in 1958 to measure the degree of people's self-disclosure. A checklist of six dimensions was used to assess participants' experience of COVID-19 related self-disclosure, including attitudes and opinions, interests and hobbies, study or work, money, personality, and body. There were 10 questions for each dimension, and each question responded to the degree of self-disclosure of four target people: father, mother, friends of the same sex, and friends of the opposite sex. A is for saying nothing to others, B is for saying something to others, C is for telling others in details. D is lying to others or revealing oneself incorrectly. A, B, C, and D were scored by 1, 2, 3, and 1 respectively, each participant had 240 scoring items and the higher the score, the higher the degree of self-disclosure. A Chinese scholar Li Linying (20) revised and translated the questionnaire, which showed good reliability and validity. In this study, the Cronbach α coefficients of the six dimensions of self-disclosure were 0.97, 0.97, 0.98, 0.98, 0.97, 0.97, and 0.99 respectively, and the overall value was 0.99.

### Peer Relationship Questionnaire

In this study, the revised version of Zou Hong's (1998) Peer Relationship Scale was adopted to investigate individuals' subjective feelings of peer relationship, including two dimensions of peer acceptance and peer fear and inferiority. There were 30 items used to assess participant's peer relationship: peer acceptance scales ranged from 1 to 20 and peer fear and inferiority scales ranged from 21 to 30. The questionnaire was scored by four points. The 30 item scales assessed Peer relationship behaviors with a 4-point scale (1 = completely inconsistent, 2 = not quite consistent, 3 = relatively consistent, 4 = completely consistent). Among them, peer acceptance subscales 1, 3, 7, 11, and 17 are a positive score, and the rest are reverse score. The higher the score, the higher the level of peer acceptance, the better the peer relationship. The subscales of peer fear and inferiority were all positive scores. The higher the score, the higher the individual felt inferiority and fear in the peer relationship, and the worse the peer relationship was. In this questionnaire, the Cronbach α coefficient of peer acceptance was 0.94, and the Cronbach α coefficient of peer fear and inferiority was 0.93.

### Loneliness Questionnaire

In this study, the UCLA Loneliness Scale developed by Russell and revised by Wang Xiangdong et al. (21) was used to measure the loneliness level of middle school students, and to investigate the subjective feeling of loneliness. A total of 20 items were used to assess participant's loneliness, which was divided into four points according to the degree of conformity: never, rarely, sometimes, and always. There were scored by 1–4 points, respectively. Among them, 1, 5, 6, 9, 10, 15, 16, 19, and 20 are reverse scores. The final scores are added up, and the loneliness scores range from 20 to 80. The higher the score is, the higher the individual loneliness level is. In this study, the Cronbach α coefficient of this questionnaire was 0.90.

### Data Analysis

In this study, Excel was first used to clean, sort, and code the data set, and then the data set was exported to SPSS.

First, descriptive statistics and correlations between the main variables were conducted. Second, to examine the relationship between COVID-19 self-disclosure and loneliness, a serial mediation was performed with COVID-19 self-disclosure as the independent variable, peer relationship as mediators in sequence, and loneliness as the dependent variable. Finally, SPSS 22.0 statistical analysis software was used to conduct variance analysis, independent sample *t*-test, Pearson correlation analysis, and simple effect analysis on the data. Confirmatory factor analysis was conducted on the data through AMOS 21.0 software and Bootstrap software to establish a structural equation model.

## Result

### Demographic Data

A total of 830 participants were included in the final statistical analysis (see Table 1). Among them, 47.5% were male and 52.05% were female. The mean age of the samples was 14.21 years old (SD = 0.82 years old), and the age range was 12–15 years old. The sample is middle school students, including 160 students in the first year of junior high school, 381 students in the second year of junior high school, and 289 students in the third year of junior high school. 12.89% of the students are only children; 43.01% are from urban areas and 56.99% are from rural areas.

**Table 1:** Demographics and responses of participants (*N* = 830).

		**Numbers**	**Percentage (%)**
Gender	Male	398	47.95
	Female	432	52.05
Age	12	28	3.37
	13	126	15.18
	14	316	38.07
	15	360	43.38
Grade	1	160	19.28
	2	381	45.90
	3	289	34.82
The one-child	yes	107	12.89
	no	724	87.21
Single parent families	yes	41	4.94
	no	789	95.06
Family location	Cities and towns	357	43.01
	rural	473	56.99
Father education	Junior high school and below	416	50.12
	High school or technical secondary school	265	31.93
	Junior College or University	137	16.51
	Master degree or above	12	1.44
Mother education	Junior high school and below	462	55.66
	High school or technical secondary school	240	28.92
	Junior College or University	120	14.46
	A graduate student	8	0.96

### Correlation Analysis of Adolescent Self-Disclosure, Peer Relationship, and Loneliness

The results of correlation analysis showed that self-disclosure was significantly positively correlated with peer acceptance, self-disclosure was significantly negatively correlated with peer fear inferiority, self-disclosure was significantly correlated with loneliness, peer acceptance was significantly negatively correlated with peer fear inferiority, and peer fear inferiority was significantly positively correlated with loneliness (see Table 2).

**Table 2:** Pearson correlation matrix of variables in Study (*N* = 830).

**Variable**	**1**	**2**	**3**	**4**
1. Self-disclosure	1			
2.Peer acceptance	0.118^**^	1		
3. Peer fear	−0.139^**^	−0.575^**^	1	
4. The loneliness	−0.173^**^	−0.744^**^	0.608^**^	1

*p < 0.05,

** *p < 0.01*,

***p < 0.001.

### Regression Analysis of Adolescent Self-Disclosure, Peer Relationship, and Loneliness

In Model 1, self-disclosure of the regression equation was taken as the independent variable and loneliness as the dependent variable. The coefficient of the influence of self-disclosure on loneliness was 0.017. In Model 1, *R*^2^ = 0.05, *P* < 0.01, adjusted *R*^2^ = 0.04, *P* < 0.01, *F* value is 4.31, and it reached a significant level. It shows that self-disclosure has a significant predictive effect on the loneliness model (see Table 3).

**Table 3:** Regression analysis of self-disclosure, peer relationship and loneliness.

**The variable name**	**Model a**	**Model 2**
**Control variables**		
Gender	−0.017	0.484
Age	1.440	0.417
Grade	0.548	−0.104
The one-child	−0.581	0.084
Single parent families	0.842	−1.685
Family location	−2.849	0.093
Father education	−0.115	0.844
Mother education	0.483	−0.755
**The independent variables**		
Self-disclosure	−0.17^**^	−0.007^**^
Company to accept		−0.545^**^
Companion fear		0.390^**^
*R*^2^	0.045.	0.610
Adjust Δ*R*^2^	0.035.	0.605
Δ*R*^2^		0.565^**^
*F*	4.313^**^	116.306^**^

** *P < 0.01*.

In Model 2, two dimensions of peer acceptance and peer fear inferiority were added on the basis of Model 1. The influence coefficients of peer acceptance of loneliness were −0.55 and 0.39 respectively. In other words, peer acceptance negatively predicted loneliness, while peer fear inferiority positively predicted loneliness. The *R*^2^ = 0.61, *P* < 0.01 in Model 2, and the *F* value is 116.31 when *R*^2^ = 0.60, *P* < 0.01, and it reached a significant level. It indicates that peer acceptance and peer fear and inferiority have significant predictive effects on loneliness model (see Table 3).

### The Mediating Effect of Adolescent Peer Relationship

Through the above correlation analysis, self-disclosure during the epidemic period was significantly correlated with peer acceptance, peer fear, inferiority, and loneliness, which met the conditions for mediating effect analysis. In addition, according to the correlation analysis, self-disclosure, peer acceptance, peer fear, and inferiority have a significant predictive effect on loneliness. Based on the above results, a structural equation model was established by AMOS 21.0 software to analyze the mediating effect of peer relationship between self-disclosure and loneliness.

**Figure 1:**
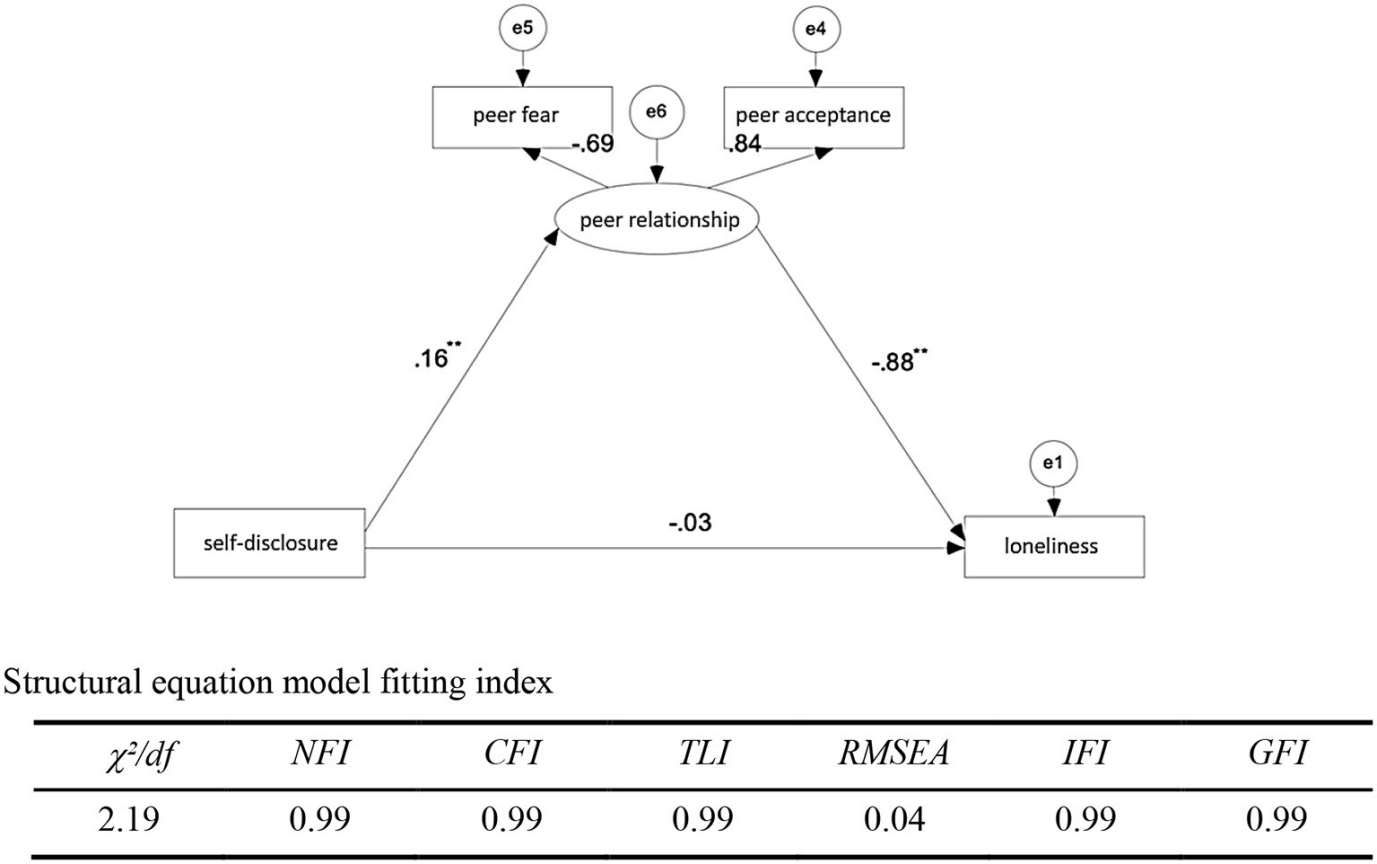
Mediating effect model of peer relationship between self-disclosure and loneliness.

Figure 1 shows that during the outbreak of self-disclosure—the path coefficient of peer relationship and peer relations—the path coefficient of loneliness have reached a significant level (*p* < 0.001); the overall effect was 0.173 (*p* < 0.01), direct effect was not significant (*p* = 0.176), the indirect effect was 0.139 (*p* < 0.01), the percentage of the mediation effect of total effect at 80.3%, and the goodness of the fit index is fairly good. It proves that the mediation effect of this model is significant, as the direct effect was not significant, so it is the partial mediating effect (see Figure 1).

## Discussion

The present study examined the relationship between COVID-19 self-disclosure, loneliness, and peer relationship in a sample of Chinese junior school students. Consistent with our hypothesis, in the period of COVID-19, adolescents' self-disclosure affected loneliness through peer relationship, that is, the level of self-disclosure can significantly predict loneliness through peer relationship, and peer relationship plays a complete mediating role.

The sudden outbreak of COVID-19 has had a great impact on human society and everyone's psychology. The adolescent period is a special period of rapid physiological and psychological changes, and it is more sensitive to external crisis events and more likely to produce various psychological crises due to the impact of the epidemic (19). Because of COVID-19, the youth have experienced home quarantine, online learning, school resumption, and exam schooling changes, among others. Those that cannot adapt to and respond to these changes could possibly enter a state of psychological crisis, and produce a series of emotional and behavioral problems, such as loneliness, anxiety, self-disclosure difficulties, and reductions in peer exchanges (22). As far as we know, this is the first study to relate adolescent's self-disclosure and loneliness during the COVID-19 outbreak. According to the results of the study, during the outbreak of COVID-19, self-disclosure was significantly positively correlated with peer acceptance, self-disclosure was significantly negatively correlated with peer fear inferiority, self-disclosure was significantly correlated with loneliness, peer acceptance was significantly negatively correlated with peer fear inferiority, and peer fear inferiority was significantly positively correlated with loneliness. According to the regression analysis and model test, we concluded that during the epidemic self-disclosure affected loneliness through peer relationship, that is, the level of self-disclosure could significantly predict loneliness through peer relationship, and peer relationship played a complete mediating role. So it validates our hypothesis. This result is consistent with previous foreign research results from Franzoi et al. (23) and Imai and Imai (15), both of which show that self-disclosure significantly affects loneliness. Researchers Feng Feng, Zhou Zongkui et al. also conducted a study on self-disclosure and loneliness in 2011, which is consistent with the results of this study. Individual self-disclosure is significantly negatively correlated with the level of loneliness, and individuals with high self-disclosure have low loneliness.

Individuals who feel a high level of social support believe that they can get help from others when they encounter difficulties and are more willing to establish good contact with others, so as to protect themselves from loneliness and negative emotions (23). Changes in self-disclosure behavior may reflect or support social reorientation, as adolescents become increasingly dependent on their peers for emotional and social support (24), and our research also indirectly demonstrates this. Adolescents with good relationships disclose more frequently to their parents and friends than others, and adolescents at this time are in the peak of peer relationship development and emotional development, and peers or parents are an important source of emotional support for adolescents. From the results of the study, we conclude that adolescents' self-disclosure affects peer relationships and further affects loneliness.

Although much research has focused on self-disclosure about peer relationships during adolescence, the medium of communication has changed dramatically over time. Social networking sites (SNS) play a prominent role, and the number of teenagers who participate in online self-disclosure has grown. Although teenagers come into contact with more strangers through social networking sites, some studies have shown that online disclosure and use of social networking sites have a greater negative impact. Research on face-to-face disclosure has mainly found positive aspects, such as increased relationships, better friendship quality, perceived greater social support, social self-esteem, and belonging (25–27). Although social networks are developing rapidly, self-disclosure through network channels have increased greatly, however, adolescents spend most time face-to face communication with teacher peers and parents in school and home, face to face interpersonal communication occupies the most section in their life. Therefore face-to face self-discloser is more conductive to adolescents overall development. Face-to-face self-disclosure is more advantageous to individual comprehensive development and helps in maintaining positive relationships.

Based on the results of this study, self-disclosure may predict happiness, and the effect of self-disclosure on well-being largely depends on the valence of induced events. Positive emotional disclosure will make people feel more positive, while negative emotional disclosure will make people feel more negative (28). Compared with pain and negative feelings, individuals are more willing to pursue the feelings of happiness and pleasure. The process of interpersonal communication is also a process of benign development, and positive self-disclosure will also indicate the good development of a partner relationship. Positive disclosure tends to occur more frequently and predict positive feedback and greater social support (29–31), which can increase feelings of connection (32). Similarly, negative disclosure may hinder others from providing public response (33). That is to say, the negative content of disclosure cannot promote the development of peer relationship and makes it difficult for individuals to respond or even produce resistance, which is not conducive to the development of peer relationships. Receiving feedback can enhance self-esteem, while lack of feedback may lead to feelings of rejection and even threaten the basic needs of the individual (34–37), resulting in loneliness and a series of negative emotions. In conclusion, the results of our study also verify the conclusions of previous studies. However, the research on the effect of self-disclosure on loneliness should not be limited to this, and more studies are needed to explain the relationship between self-disclosure and loneliness.

Because of its reach and ferocity, COVID-19 has been characterized as a once in a century pandemic; however, it is not the first pandemic of the modern era. SARs, AIDS, Ebola, and more have struck across the globe, each presenting a risk to public health and limiting how people interact with each other (6). This quarantine particularly has changed adolescent's lifestyle, the decrease of activity space and direct communication may result in communication disorders, loss of interest, fear, tension, anxiety, loneliness, and other negative psychological problems. Understanding the influence of novel coronavirus pneumonia, particularly during COVID-19, and how self-disclosure affects loneliness through peer relationships informs our approach to resolve adolescent's psychological problems. For instance, we suggest that research and practice need to revisit commonly held assumptions about self-disclosure, and what is considered appropriate and necessary to self-disclose. By understanding pandemic-related self-disclosures, we believe researchers will be able to better study the relationships between self-disclosure and loneliness during health emergencies.

## Limitations and Future Research Directions

This study has some important limitations due to sampling techniques. Firstly, this study randomly selected junior high school students in Gansu and Shandong provinces, and only targeted adolescents aged 12–15. Future research can investigate self-disclosure levels of adolescents in different regions, and study different perspectives such as culture, region, ethnicity, and age. Secondly, since we do not have pre-COVID-19 data and this study was a cross-sectional study, the findings obtained cannot infer that the COVID-19 pandemic caused the effects of adolescent self-disclosure on loneliness and peer relationships. Finally, the study was conducted among junior high school students in China and is not representative of other global groups. Therefore, a longitudinal study of different groups is needed. In addition, this research also has certain educational significance. In order to have good mental health development before the arrival of adulthood, and develop a healthy personality and study and live smoothly, families, schools, and individuals should attach importance to adolescents' self-disclosure, help them to adjust their negative emotions, and reveal themselves rationally. Adolescents should learn to seek support and help from others to reduce their negative emotions, stress, and anxiety.

## Conclusion

This study focused on factors associated with self-disclosure, loneliness, and peer relationships among adolescents during the COVID-19 pandemic. Therefore, it can be concluded that in the period of COVID-19, adolescents' self-disclosure affects loneliness through peer relationships, that is, the level of self-disclosure can significantly predict loneliness through peer relationships, and peer relationships play a complete mediating role. This helped to educate people about the importance of self-disclosure during the COVID-19 pandemic, enabling adolescents to approach the epidemic in a positive way. Mental health education and consultation schemes should be implemented to prevent and alleviate psychological problems associated with COVID-19, particularly among adolescents.

## Data Availability Statement

The raw data supporting the conclusions of this article will be made available by the authors, without undue reservation.

## Ethics Statement

The studies involving human participants were reviewed and approved by Institutional Review Board at Northwest Normal University, development of Psychology. Written informed consent to participate in this study was provided by the participants' legal guardian/next of kin.

## Author Contributions

BH conceived the present study, collected the data, and conducted the statistical analyses. RC drafted the manuscript. LC supervised the study and helped to revise the manuscript. All authors read and approved the final manuscript.

## Conflict of Interest

The authors declare that the research was conducted in the absence of any commercial or financial relationships that could be construed as a potential conflict of interest.

## Publisher's Note

All claims expressed in this article are solely those of the authors and do not necessarily represent those of their affiliated organizations, or those of the publisher, the editors and the reviewers. Any product that may be evaluated in this article, or claim that may be made by its manufacturer, is not guaranteed or endorsed by the publisher.
